# Case of ST-Elevation Myocardial Infarction in a 32-Year-Old Male Receiving Bleomycin, Etoposide, and Cisplatin Chemotherapy for Embryonal Carcinoma

**DOI:** 10.7759/cureus.40089

**Published:** 2023-06-07

**Authors:** Emmanuel Bassil, Harjinder Singh, Omair Ahmed, Shamik Parikh

**Affiliations:** 1 Internal Medicine, Henry Ford Allegiance, Jackson, USA

**Keywords:** acute stemi, embryonal cell carcinoma, cisplatin-induced cardiotoxicity, bleomycin side effect, st-elevation myocardial infarction (stemi)

## Abstract

Myocardial infarction in young individuals has unique risk factors compared to the older population. Along with usual risk factors, one should explore causes such as recreational drug use, medication-induced myocardial infarction, and spontaneous coronary artery dissection. Here, we present the case of a 32-year-old male who presented with chest pain and was found to have complete thrombotic occlusion of the right coronary artery. He recently started receiving chemotherapy with bleomycin, etoposide, and cisplatin (PEB). In the absence of other risk factors and previous reports of similar cardiotoxicity with bleomycin, the patient was deemed to have an adverse effect from the chemotherapy regimen.

## Introduction

Bleomycin is an alkylating agent that belongs to the class of cytotoxic agents known as anti-tumor antibiotics. It is a frequent component of several chemotherapeutic regimens for a range of neoplasms including lymphomas, ovarian, cervical, and testicular cancers. Among its side effects, it is mostly known to cause pulmonary fibrosis in patients; however, a growing body of literature sheds light on its potential for cardiotoxicity in those undergoing therapy with this agent. A report has documented its role in causing pericarditis [1] and acute-onset substernal chest pain without any long-term sequelae [2]. It is interesting to note that a certain subset of patients, particularly young males undergoing treatment for a testicular germ cell tumor with a bleomycin-containing regimen, are more predisposed to coronary artery disease and increased incidence of myocardial ischemic events, complications not typically seen in healthy patients in this age group [3-5]. We present a case of a young patient with no prior cardiac history who suffered a myocardial infarction (MI) after treatment with the bleomycin, etoposide, and cisplatin (PEB) regimen. 

## Case presentation

A 32-year-old male presented to the ED with worsening substernal chest pain radiating to the left shoulder accompanied by nausea and shortness of breath. Past history was significant for Stage 3A (pT2, pN1, cM1a, S1) embryonal carcinoma of the left testicle for which the patient underwent left radical orchiectomy and was currently undergoing cycle 1 of chemotherapy with a regimen including PEB due to the presence of paraaortic lymphadenopathy and presence of multiple suspicious pulmonary nodules. He had received bleomycin 24 hours prior and cisplatin five days before the presentation.

**Figure 1:**
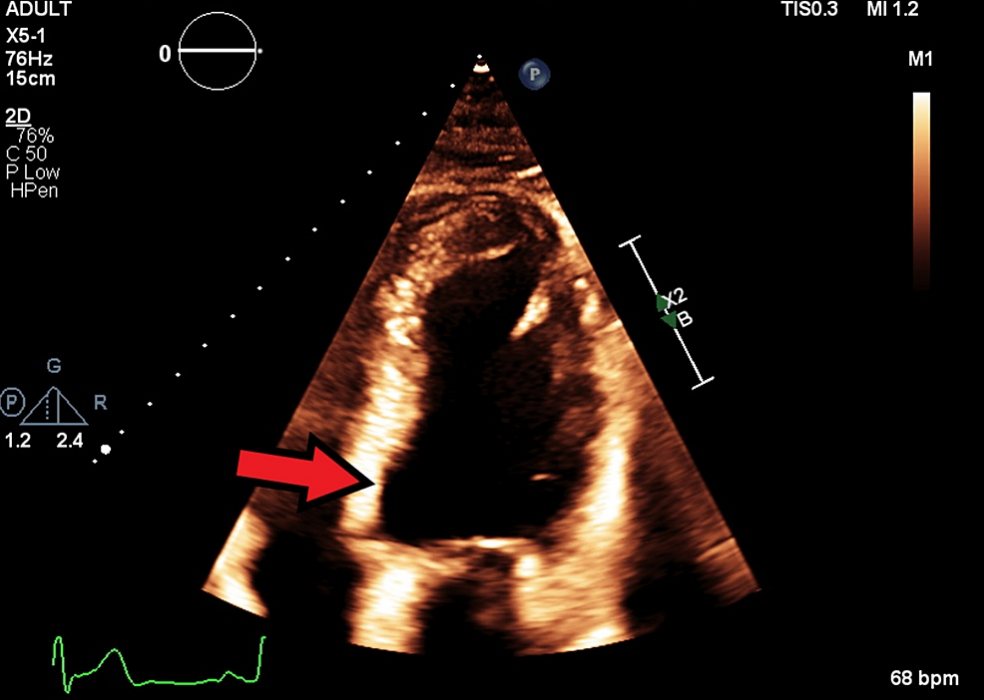
Image from baseline echocardiogram prior to treatment revealed normal ejection fraction (EF) of 61%, normal ventricular cavity size and wall thickness, and no valvular or wall motion abnormalities.

A baseline echocardiogram (Figure 1) before therapy initiation revealed a normal ejection fraction (EF) of 61%, normal ventricular cavity size and wall thickness, and no valvular or wall motion abnormalities. The patient had no history of heart disease but reported a 10-pack-year history of smoking and stage I hypertension. He had a history of coronary artery disease in his father and both paternal grandparents. Pulmonary function tests were within normal limits.

**Figure 2:**
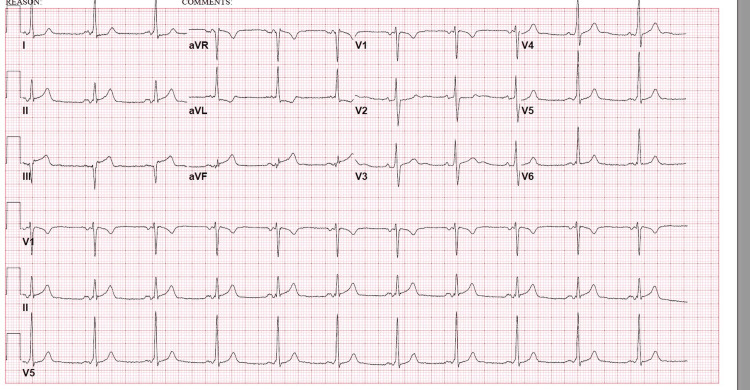
EKG on presentation revealed ST elevation in the inferior leads suggestive of STEMI. STEMI, ST-elevation myocardial infarction.

He presented to the ED the day after receiving cycle 1, day 9 of bleomycin reporting a one-day history of worsening pressure-like left-sided chest pain accompanied by nausea and shortness of breath. An initial ECG showed ST elevations in leads III and aVF (Figure 2). High-sensitivity troponin was 1,267 ng/dL (normal < 19). He underwent emergent cardiac catheterization, which revealed 100% thrombotic occlusion at the proximal right coronary artery as well as faint collaterals from the left-to-right system. There was no evidence of atherosclerotic disease or stenosis in any other vessels. Thrombectomy was performed, followed by stent placements for significant residual thrombus. Post-procedure angiography revealed 0% residual stenosis.

**Figure 3:**
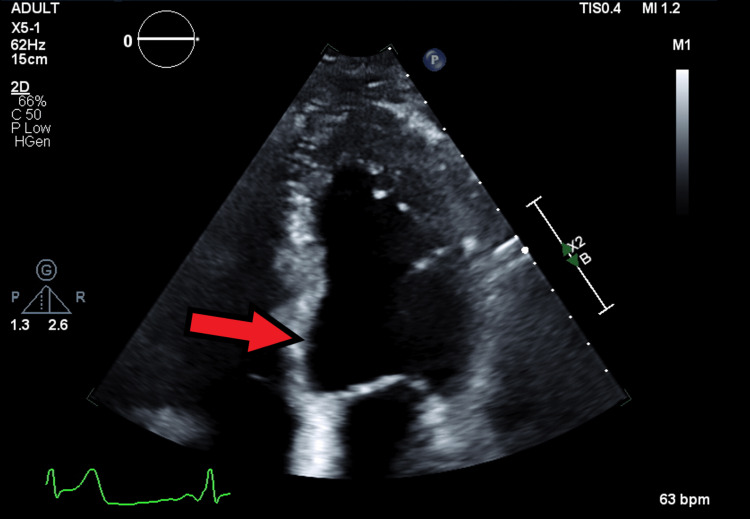
Still of post-procedure echocardiogram; EF of 55%, normal left ventricle size and thickness, and hypokinesis of the basal-mid inferior and inferoseptal wall. EF, ejection fraction.

Post-procedure echocardiogram (Figure 3) revealed EF of 55%, normal left ventricle size and thickness, and hypokinesis of the basal-mid inferior and inferoseptal wall. Following this event, bleomycin was stopped and his chemotherapy regimen was changed to etoposide and cisplatin. On a three-month follow-up, the patient was tolerating the new regimen well and is reporting no post-MI complications.

## Discussion

MI in young patients <45 years old is uncommon, especially with no family history of heart disease [6]. It is important to note that our patient had some risk factors for early MI, including male gender and smoker status [7]. However, the proximity of his presentation to his chemotherapy cycle makes the BEP regimen a likely cause of the STEMI. This is further reinforced by the lack of atherosclerosis and faint collaterals on angiography, suggesting an acute thrombus. It is important to note, however, that no optical coherence tomography (OCT) was done to conclusively determine the cause of the STEMI which is a limitation of our case. While a prospective study has established that patients treated with chemotherapy for testicular cancer had a higher incidence of CAD and MI during long-term follow-up [8], there is little data on acute MIs during early treatment.

Testicular cancer is the most common cancer in men between 15 and 44 years of age [9]; however, most of the data on MIs in this population are reported in the older age range of these patients. The relative rarity of similar case reports in patients between 15 and 30 suggests that age might increase the risk of BEP-induced MI.

The literature reports 13 cases of acute BEP-induced MI (Table 1) in patients under 45, an uncommon side effect. However, it is unclear which drug in the regimen is associated with MIs. Cisplatin’s association with vascular side effects, especially venous thromboembolism, is well reported in the literature [10-12]. However, a meta-analysis assessing the incidence of arterial thromboembolism specifically secondary to cisplatin found no significant increase compared to non-cisplatin chemotherapy [13]. Moreover, while there is evidence of cisplatin causing vascular injury through the detection of elevated von Willebrand factor (vWF) [14], more research is required to establish that this translates to a higher risk of arterial thromboembolism, MI, and cerebrovascular accidents. Cisplatin has also been linked to ototoxicity, nephrotoxicity, neurotoxicity, hypomagnesemia, myelosuppression, and gastrointestinal toxicity [15].

**Table 1:** List of case reports of post-PEB chemotherapy leading to MI LCx: left circumflex; RCA: right coronary artery; LAD: Left anterior descending; prox: proximal; PEB: cisplatin, etoposide, bleomycin; VBP: vinblastine, bleomycin, cisplatin.

	Age, sex	Cancer	Therapy	Duration	Artery	Reference
1	33/M	Testicular seminoma	PEB	End of 1^st^ cycle	LCx	[16]
2	30/M	Testicular seminoma	PEB	After 3 cycles	LAD	[17]
3	34/M	Testicular seminoma	PEB	Cycle 1, Day 5	RCA	[18]
4	30/M	Embryonal carcinoma	PEB	Cycle 3, Day 9	RCA	[19]
5	38/M	Testicular seminoma	PEB	Cycle 4, Day 10	Prox. LAD	[20]
6	34/M	Embryonal carcinoma with a minor component of choriocarcinoma	PEB	Cycle 2, Day 7	LAD	[20]
7	37/M	Retroperitoneal non-seminomatous germ cell tumor	PEB	Cycle 2, Day 10	LAD	[21]
8	36/M	Embryonal carcinoma	PEB	Cycle 1, Day 9	RCA	[22]
9	27 /M	Testicular seminoma	PEB	Cycle 2, Day 8	Prox. LAD	[23]
10	36/M	Immature teratoma	PEB	After 2 cycles	LCX, LAD	[24]
11	24/M	Embryonal carcinoma	VBP	18 months after the 6^th^ course	LAD	[25]
12	33/M	---	PEB	6 weeks after the start of therapy		[26]

It is important to note that it is difficult to ascertain whether this MI was caused by bleomycin, cisplatin, or a combination of both, as bleomycin is usually given in combination with cisplatin. In our patient, bleomycin was discontinued while he was continued on etoposide and cisplatin, which he tolerated well over the next three months. His good response to the continuation of cisplatin, as well as emerging evidence that cisplatin alone cannot explain chemotherapy-induced MIs, leads us to believe that bleomycin may have played a role in the pathogenesis of his MI.

Bleomycin is usually known for pulmonary toxicity [27], while little is known about the mechanism of bleomycin-induced MI. There are reports of bleomycin causing thrombotic microangiopathy [28] and vascular stenosis [29], but this has not been studied in population-based trials or randomized controlled trials and the mechanism remains unknown.

## Conclusions

In this case of PEB-associated MI, bleomycin’s role could either be potentiating cisplatin’s vascular toxicity, and the patient's pre-existing risk factors, or could be acting through a different independent mechanism. Nonetheless, this case and others like it show the importance of minimizing every risk factor in patients about to start PEB chemotherapy, even in relatively young patients. Of course, more research must be done to establish a causal link for either cisplatin or bleomycin in the pathogenesis of early MI.
